# Histopathologic Profile of Salivary Gland Tumors among Specimens from a Tertiary Care Hospital: A Descriptive Cross-sectional Study

**DOI:** 10.31729/jnma.4898

**Published:** 2020-10-31

**Authors:** Dilasma Ghartimagar, Arnab Ghosh, Manish Kiran Shrestha, Sushma Thapa, Om Prakash Taiwar

**Affiliations:** 1 Department of Pathology, Manipal College of Medical Science, Pokhara, Nepal; 2 Department of Radiology, Charak Memorial Hospital, Pokhara, Nepal

**Keywords:** *mucoepidermoid carcinoma*, *pleomorphic adenoma*, *salivary gland*, *tumor*

## Abstract

**Introduction:**

Salivary gland tumors are rare, comprising about 3% of all head and neck neoplasms. About 80% of the tumors are in parotids, 10% in submandibular glands and the remainders are distributed in sublingual and minor salivary glands. This study was conducted to evaluate the relative frequencies, types, site of distribution and the histopathological features of salivary gland tumors.

**Methods:**

A descriptive cross-sectional study was conducted in the Department of Pathology, Manipal College of Medical Sciences, Pokhara from January 2011 to December 2019. Ethical approval was taken from the institutional review committee of Manipal College of Medical Sciences (Ref: 314). Convenient sampling was done among specimen. Data were entered in Microsoft Excel and analyzed using Statistical Package for the Social Sciences version 21.

**Results:**

Among the 130 specimens, the patients' age ranged from 6 to 78 years with a mean age of 37.26 years for benign tumors and 48.45 years for malignant tumors. There was female predominance with a male to female ratio of 1:1.36. There were 98 cases of benign tumors, commonest being pleomorphic adenoma with 82 (83.67%) cases which was noticed more frequently in fourth decade of life. Among the 32 malignant tumors, mucoepidermoid carcinoma was the commonest tumor 20 (62.5%), followed by adenoid cystic carcinoma 7 (23.33%).

**Conclusions:**

Benign salivary gland tumors were more common than the malignant tumors and the most common site of location was the parotid for both the benign and malignant tumors. Female outnumbered the male population in benign tumors whereas males were slightly more than females in malignant tumors. This study corroborated well with other previously published studies.

## INTRODUCTION

Salivary gland tumor is rare and comprises a wide variety of benign and malignant neoplasms which shows different biological behavior. There are three major salivary glands, namely parotid, submandibular and sublingual along with other minor salivary glands that are distributed in the oral cavity mucosa. Both neoplastic and non-neoplastic diseases may develop within any of these sites.^1,2^

Salivary gland tumor comprises about 3% of all head and neck tumors. Benign tumors are more common when compared to malignant tumors. In the parotid gland, about 80% of the tumors are benign, whereas in the submandibular gland it drops to 60% and in the oral cavity, malignant salivary gland tumors outnumber the benign tumors.^3,4^

This study was done to evaluate the relative frequencies, age at the occurrence, sex ratio, site of distribution, and the histopathological features of salivary gland tumors.

## METHODS

A descriptive cross-sectional study was conducted in the Department of Pathology, Manipal Teaching Hospital, Pokhara for a duration of Jan 2011 to Dec 2019. Ethical approval was taken from the institutional review board of Manipal College of Medical Sciences (Ref: 314). The study included all the cases of salivary gland neoplasm irrespective of their sites that had undergone surgery in the hospital. Non-neoplastic lesions which include inflammatory disorder of infectious and granulomatous etiology were not included in the study. The specimens were received for histopathological examination from the institution. The sample size was calculated as,

$$\begin{array}{ll} \text{n} & \text{= }{\text{Z}}^{\text{2}}\times \text{p}\times \text{(1}-\text{p)}/{\text{e}}^{\text{2}}\\ & \text{= }1.96^{\text{2}}\times (0.5)\times (1-0.5)/{\left(0.09\right)}^{\text{2}}\\ & \text{= }118.56 \end{array}$$

Where,

n = Sample size
Z = 1.96 at 95% confidence interval
p = population proportion, 50%
e = margin of error, 9%

Taking a 10% non-response rate, the final sample size was calculated as 130.41. Therefore, 130 specimens were taken in the study.

Relevant clinical history along with the site of the lesions was analyzed. The salivary gland tumors were routinely fixed in 10% formalin. Large specimens were cut for proper fixation before grossing. After fixation, adequate sections were given from the tumor, capsules, margins, and base. The slides were routinely stained with hematoxylin and eosin (H & E) stains and also with special stains wherever it was required. The slides were analyzed by two pathologists for a definite diagnosis.

The data were analyzed using Microsoft Excel and Statistical Package for the Social Sciences (SPSS) 21.0 version.

## RESULTS

A total of 130 cases of salivary gland tumors have been reported in the same period. There were 55 males and 75 females with a male to female ratio of 1:1.36. Age ranged from 6 to 78 years with a mean age of 40.01 years. The mean age for benign tumors was 37.26 years and for malignant tumors was 48.45 years. There were 98 (75.38%) benign tumors and 32 (24.61%) malignant tumors. Male had 36 (27.69%) benign tumors and 19 (14.61%) malignant tumors whereas female had 62 (47.69%) benign tumors and 13 (10%) malignant tumors. The most common site of the tumor was parotid 89 (68.46%) which was followed by submandibular gland 18 (13.84%). Parotid gland showed mostly benign tumors with 68 (52.30%) cases and malignant tumor with 21 (16.15%) cases; submandibular gland showed 14 (10.76%) cases of benign tumors and 4 (3.07%) cases of malignant tumors; sublingual gland showed only 8 (6.15%) cases of benign tumors and other minor salivary glands showed 8 (6.15%) cases of benign tumors and 7 (5.38%) cases of malignant tumors.

Among the 98 (75.38%) benign neoplastic conditions, pleomorphic adenoma (PA) was the commonest with 82 (83.67%) cases followed by Warthin tumor with 8 (8.16%) cases and 2 (2.04%) cases each of monomorphic adenoma, neurofibroma, and schwannoma (Table 1).

**Table 1:** Benign salivary gland tumors with their type and age distribution. (n = 98)

Benign Salivary Gland Tumors	Cases	Age-wise distribution							
	n (%)	<10	11-20	21-30	31-40	41-50	51-60	61-70	>70
Pleomorphic adenoma	82 (83.67)	2 (2.04)	10 (10.2)	20 (20.4)	22 (22.44)	14 (14.28)	10 (10.20)	2 (2.04)	2 (2.04)
Monomorphic ademona	2 (2.04)	-	-	-	1 (1.02)	-	1 (1.02)	-	-
Warthin tumor	8 (8.16)	-	1 (1.02)	-	-	1 (1.02)	2(2.04)	-	4(4.08)
Benign lymphoepithemioma	1 (1.02)	-	-	-	-	-	1 (1.02)	-	-
Neurofibroma	2 (2.04)	-	-	-	-	1(1.02)	1(1.02)	-	-
Schwannoma	2 (2.04)	-	-	-	1(1.02)	1(1.02)	-	-	-
Lipoma	1 (1.02)	-	-	-	-	-	1(1.02)	-	-
Total	98 (100)	2 (2.04)	11 (11.22)	20 (20.40)	24 (24.48)	17 (17.34)	16 (16.32)	2 (2.04)	6 (6.12)

Pleomorphic adenoma and Warthin tumors were found more in females with 50 and 7 cases respectively. The site of tumor location was also more in the parotid for both benign and malignant tumors. Among the benign tumors, 54 cases of PA were in the parotid, 12 cases were in the submandibular gland and 8 cases each were in the sublingual and other minor salivary glands like the palate, cheek, lip, nasal cavity, etc. There were two cases of monomorphic adenoma, both of which were in the submandibular gland. There were eight cases of Warthin tumor and all the cases showed the tumor in the parotid gland (Table 2).

**Table 2:** Benign salivary gland tumors: type, gender, and site distribution (n = 98)

Benign Salivary Gland Tumors	Cases n (%)	Sex		Site			
		Male	Female	Parotid	Sub mandibular	Sub lingual	Other/minor
Pleomorphic adenoma	82 (83.67)	32 (32.65)	50 (51.02)	54 (55.10)	12 (12.24)	8 (8.16)	8 (8.16)
Monomorphic adenoma	2 (2.04)	-	2 (2.04)	-	2 (2.04)	-	-
Warthin tumor	8 (8.16)	1 (1.02)	7 (7.14)	8 (8.16)	-	-	-
Benign lymphoepithelioma	1(1.02)	-	1(1.02)	1 (1.02)	-	-	-
Neurofibroma	2(2.04)	1(1.02)	1(1.02)	22 (2.04)	-	-	-
Schwannoma	2(2.04)	1(1.02)	1(1.02)	2 (2.04)	-	-	-
Lipoma	1(1.02)	1(1.02)	-	1 (1.02)	-	-	-
Total	98 (100)	36 (36.73)	62 (63.26)	68 (69.38)	14 (14.28)	8 (8.16)	8 (8.16)

Pleomorphic adenoma was seen in all age groups and decade of life with 20 (24.39%) cases while the 4th the maximum number of cases was seen in the 3rd decade of life had 22 (26.82%) cases (Table 3).

**Table 3:** Pleomorphic adenoma correlation between age, gender and site (n = 82)

Age (yrs)	Number (%)	Gender		Site			
		Male	Female	Parotid	Sub mandibular	Sub lingual	Other/minor
<10	2 (2.43)	0	2 (2.43)	2 (2.43)	0	0	0
11-20	10 (12.19)	3 (3.65)	7 (8.53)	8 (9.75)	1 (1.21)	0	1 (1.21)
21-30	20 (24.39)	8 (9.75)	12 (14.63)	12 (14.63)	3 (3.65)	3 (3.65)	2 (2.43)
31-40	22 (26.82)	10 (12.19)	12 (14.63)	14 (17.07)	3 (3.65)	2 (2.43)	3 (3.65)
41-50	14 (17.07)	6 (7.31)	8 (9.75)	9 (10.97)	3 (3.65)	1 (1.21)	1 (1.21)
51-60	10 (12.19)	3 (3.65)	7 (7.31)	7 (7.31)	1(1.21)	1 (1.21)	1 (1.21)
>60	4 (4.87)	2 (2.43)	2 (2.43)	2 (2.43)	1 (1.21)	1 (1.21)	0
Total	82 (100)	32 (42.68)	50 (60.97)	54 (65.850	12 (14.63)	8 (9.75)	8 (9.75)

Warthin tumor was seen more in the age group of above 70 years with 4 (50%) cases while 2 (25%) cases were seen in the 6th decade of age. Here also, females showed more Warthin tumors with 7 (87.5%) cases while the male showed only 1(12.5%) case (Table 4) and this tumor was noticed both in major and minor salivary glands.

**Table 4:** Warthin tumor correlation with age, site, and gender (n = 8)

Age group	Cases n (%)	Site				Sex	
		Parotid	Submand.	Subling.	Others / minor	Male	Female
11-20	1 (12.5)	1 (12.5)	0	0	0	0	1 (12.5)
41-50	1(12.5)	1 (12.5)	0	0	0	0	1 (12.5)
51-60	2 (25)	2 (25)	0	0	0	1	1 (12.5)
>70	4 (50)	4 (50)	0	0	0	0	4 (50)
Total	8 (100)	8 (100)	0	0	0	1(12.5)	7 (87.5)

Out of 32 cases of malignant tumors, mucoepidermoid carcinoma (MEC) was the commonest with 20 (62.5%) cases and it was noticed in the age ranging from 12 to 75 years. This tumor was more in of 6th decade of life with 6 (18.75%) cases while the 7th decade showed 4 (12.5%) cases of MEC (Table 5).

**Table 5:** Malignant salivary gland tumors - type and age distribution. (n = 32)

Malignant Salivary Gland Tumors	Cases n (%)	Age groups					
		11-20	21-30	31-40	41-50	51-60	61-70	>70
Mucoepidermoid	20 (62.5)	2 (6.25)	2 (6.25)	-	3 (9.37)	6 (18.75)	4 (12.5)	3 (9.37)
Adenoid cystic	7 (21.8)	-	-	-	2 (6.25)	1 (3.12)	2 (6.25)	2 (6.25)
Malignant mixed tumor	1 (3.12)	-	-	-	-	1 (3.12)	-	-
Lymphoepithelial carcinoma	1 (3.12)	-	-	-	-	1 (3.12)	-	-
Epithelial myoepithelial carcinoma	1 (3.12)	-	-	-	-	-	1(3.12)	-
NHL	2 (6.25)	-	-	-	-	-	1(3.12)	1(3.12)
Total	32(100)	2(6.25)	2(6.25)	-	5(15.62)	9(28.12)	8(25)	6(18.75)

There were 7 (21.87%) cases of adenoid cystic carcinoma where three cases were seen in males and four cases were seen in females. There was also one case of epithelial myoepithelial tumor and two cases of Non-Hodgkins Lymphoma. Mucoepidermoid carcinoma was only the tumor which showed male predominance with 15 cases while female showed only five cases of this tumor. Coming to the site of distribution, MEC was seen more in the parotid gland with 13 (65%) cases, followed by minor salivary glands with 5 (25%) cases.

## DISCUSSION

The salivary gland may give rise to various neoplastic conditions and more than 30 different types of neoplastic entities have been reported. Though salivary gland tumors are uncommon, parotid is the commonest site of location for both benign and malignant tumors.^5^ Present study showed 98 cases of benign tumors and 32 cases of malignant tumors. In both the benign and malignant tumors, parotid was the most common site of location for both benign and malignant tumors with 68 (52.30%) benign cases and 21 (16.15%) malignant cases. Like our study, Musani MA, et al. showed 74.5% of benign tumors and 25.5% of malignant tumors in the parotid.^6^ Similarly, Shrestha S, et al. from Nepal showed the parotid being the most common site of tumor location with 52 (29.54%) cases of benign tumors and 72 (40.90%) cases of malignant tumors. They had more malignant cases than benign cases as their study was done in the cancer center but we had more benign cases in the parotid glands.^7^ Another study from Nepal also showed 44 (86%) cases of benign tumors and 7 (14%) of malignant tumors in the parotid.^8^ After the parotid gland, the submandibular gland is the second most common site for the salivary gland tumor location. Jain S, et al, Bjorndal K, et al., and Nepal A, et al. showed the submandibular gland as the second commonest location with 30 (26.7%), 116 (12.2%), and 9 (17%) cases respectively.^5,8,9^ Present study also showed 18 (10.76%) cases in the submandibular region with 14 benign cases and four malignant cases.

Musani MA, et al. in their study found the highest number of benign tumors in the 4th decade and malignant tumors in the 5th decade.^6^ Shrestha S, et al. in their study showed benign salivary gland tumors being more common in the 4th decade and the peak age incidence for malignant salivary gland tumors was 5th decade.^7^

Ahrnad S, et al. in their study showed the highest incidence for a benign tumor in the 3rd and 4th decade and malignant tumors in the 4th and 5th decades of life.^1^ Rachakonda S, et al. showed the peak incidence of most of the benign tumors in the 4th and 5th decade and malignant tumors in the 6th and 7th decade of life.^10^ Present study also showed, the maximum number of benign tumors in the 3rd and 4th decade while the malignant tumors were in the 6^th^ and 7th decade of life. Several published studies have reported more frequent involvement of tumors in females than males.^5,6,11–13^ Some other studies showed the male predominance in salivary gland tumors.^1,7,14^ Present study showed more number of tumors in females than in males with a male to female ratio of 1:1.36.

Pleomorphic adenoma is the most common salivary gland tumor in both children and adults, accounting for the majority of all salivary gland neoplasms. This tumor occurs in all ages but is most common in the third to sixth decades of life. The average age of presentation is approximately 45 years with a female-to-male ratio of 2:1.^15^ In the present study, PA was the commonest pathology with 82 (83.67%) cases followed by Warthin tumor with 8 (8.16%) cases as most other studies also had the similar findings.^5–8,14^ Present study also noted that PA was most commonly noted from 2nd to 6th decades of life and with male to female ratio of 1:1.64.

A monomorphic adenoma is a rare tumor and accounts for 1.5% of all tumors in the major and minor salivary glands.^15^ Present study also showed 2 (1.53%) cases of monomorphic adenoma in the submandibular gland and both the cases were in female patients. Major salivary gland benign soft tissue neoplasms include neural tumors, most frequently neurofibroma or schwannoma, and these are thought to arise from the facial nerve radicals.^16,17^ Musani MA, et al. showed 3 (02%) cases of neurofibroma and 4 (02.6%) cases of schwannomas in their study.^6^ We reported 2 (1.53%) cases each of neurofibroma and schwannoma and all of these tumors were in the parotid gland.

Among the malignant tumors, mucoepidermoid carcinoma was the most widely recognized malignant tumor with 20 (62.5%) cases, followed by adenoid cystic carcinoma with 7 (21.87%) cases in the present study. Out of 20 cases of MEC, we observed 13 cases of MEC in the parotid gland. Similarly, other published studies also showed MEC as the commonest malignant tumors in the parotid region.^6,18–20^ Adenoid cystic carcinomas comprise about 10% of all epithelial salivary neoplasms and most frequently involve the parotid gland.^21^ Some studies have shown Adenoid Cystic Carcinoma as the most common malignant tumor of the parotid gland.^1,5,8,22,23^ We had only three cases of adenoid cystic carcinoma in the parotid gland out of seven cases. Carcinoma-ex pleomorphic adenoma has summarized that they comprise approximately 3.6% of all salivary tumors and 12% of all salivary malignancies.^24^ Musani MA, et al. Nepal A, et al. and Shrestha A, et al. in their studies have reported Carcinoma-ex pleomorphic adenoma to be 3 (5.76%) cases, 2 (4%) cases, and 6 (5.4%) cases respectively.^6–7^ Present study had only 1 (3.12%) case of Carcinoma-ex pleomorphic adenoma.

Epithelial myoepithelial carcinoma represents around 1% of the salivary gland tumors and is more common in a female with a peak incidence in the 6th and 7th decades.^25^ Present study also showed one case of this tumor in female patients of age 65 years in the right parotid gland. Lymphoepithelial carcinoma of the salivary gland is a rare tumor and accounts for less than 1% of all salivary gland tumors.^26^ This tumor shows a slight female predominance and a higher frequency of parotid gland involvement. Bjorndal K et al in their study period of 15 years showed lymphoepithelial carcinoma to be 20 (2.1%) in the parotid gland and 5 (0.5%) in the submandibular gland.^9^ We had only 1 (0.76%) case of lymphoepithelial carcinoma in the parotid region of 56 years old female patient. Non-Hodgkin lymphomas occurring as salivary gland tumors are uncommon which comprises about 2% of all salivary gland tumors.^27^ Musani MA, et al. showed 2 (3.84%) cases of lymphoma in their study.^6^ We also had 2 (1.53%) cases of lymphoma, one was in the 7th decade and the other was in the 8th decade of life and both had tumors in the parotid gland.

## CONCLUSIONS

This study concluded that pleomorphic adenoma was the commonest benign salivary gland tumors with a female predominance and among malignant tumors; mucoepidermoid carcinoma was the commonest with a male predominance. Benign tumors were noticed more in the 3rd and 4th decade of life whereas malignant tumors were seen more in the 6th and 7th decade of life.

## Conflict of Interest

**None.**
